# Prepregnancy Emergency Department Use and Risks of Severe Maternal and Neonatal Morbidity in Canada

**DOI:** 10.1001/jamanetworkopen.2022.29532

**Published:** 2022-09-02

**Authors:** Catherine E. Varner, Alison L. Park, Joel G. Ray

**Affiliations:** 1 Schwartz/Reisman Emergency Medicine Institute, University of Toronto, Toronto, Ontario, Canada; 2 Department of Emergency Medicine, Mt Sinai Hospital, Toronto, Ontario, Canada; 3 Department of Family & Community Medicine, University of Toronto, Toronto, Ontario, Canada; 4 ICES, Toronto, Ontario, Canada; 5 Keenan Research Centre, Li Ka Shing Knowledge Institute, St Michael’s Hospital, Toronto, Ontario, Canada; 6 Departments of Medicine and Obstetrics and Gynecology, St Michael’s Hospital, Toronto, Ontario, Canada

## Abstract

**Importance:**

Emergency department (ED) use during pregnancy may be associated with worse obstetrical outcomes, possibly because of differences in access to health care. It is not known whether ED use before pregnancy is associated with serious adverse maternal and perinatal outcomes.

**Objective:**

To study the association between prepregnancy ED use and adverse maternal and perinatal outcomes.

**Design, Setting, and Participants:**

This population-based cohort study was conducted in Ontario, Canada, and included all livebirths and stillbirths from April 2003 to January 2020.

**Exposures:**

Main exposure was any ED encounter within 90 days preceding the start of the index pregnancy.

**Main Outcomes and Measures:**

Primary outcome was a composite of severe maternal morbidity (SMM) from 20 weeks’ gestation to 42 days’ post partum. Secondary outcomes included severe neonatal morbidity (SNM) from 0 to 27 days, neonatal death, and stillbirth. Relative risks (RRs) were adjusted for maternal age, income, and rurality.

**Results:**

Of 2 130 245 births, there were 2 119 335 livebirths (99.5%) and 10 910 stillbirths (0.5%). The mean (SD) maternal age was 29.6 (5.4) years, 212 478 (9.9%) were rural dwelling, and 498 219 (23%) had 3 or more comorbidities. Among all births, 218 011 (9.7%) had a prepregnancy ED visit. The rate of SMM was higher among women with a prepregnancy ED visit than those without (22.3 vs 16.5 per 1000 births), with an RR of 1.34 (95% CI, 1.30-1.38) and an adjusted RR (aRR) of 1.37 (95% CI, 1.33-1.42). Compared with no prepregnancy ED visit, the aRR was higher in those with 1 (1.29; 95% CI, 1.24-1.34), 2 (1.51; 95% CI, 1.42-1.61), and 3 or more (1.74; 95% CI, 1.61-1.90) ED visits. Prepregnancy ED visits for a hematological (aRR, 13.60; 95% CI, 10.48-17.64), endocrine (aRR, 4.96; 95% CI, 3.72-6.62), and circulatory (aRR, 2.27; 95% CI, 1.68-3.07) conditions were associated with the highest aRRs for SMM. The rate of SNM was higher among newborns whose mother visited the ED within 90 days before pregnancy (68.2 vs 55.4 per 1000 births; aRR, 1.24; 95% CI, 1.22-1.26) as was the risk of neonatal death (aRR, 1.26; 95% CI, 1.16-1.37) and stillbirth (aRR, 1.18; 95% CI, 1.11-1.25).

**Conclusions and Relevance:**

In this study, ED use was common before pregnancy. These findings suggest that ED use may not only reflect a woman’s access to prepregnancy care but also higher future risk of severe maternal and perinatal morbidity, potentially offering a useful trigger for health system interventions to decrease adverse pregnancy outcomes.

## Key Points

**Question:**

Is there an association between emergency department (ED) use before pregnancy and risk of severe maternal and neonatal morbidity?

**Findings:**

In this cohort study of more than 2 million births, outcomes were compared between 218 011 patients with an ED visit 90 days before the start of a pregnancy with 1 912 234 without an ED visit. Prepregnancy ED use was associated with a higher risk of severe maternal morbidity, severe neonatal morbidity, stillbirth, and neonatal death, especially as the number of ED visits increased.

**Meaning:**

These findings suggest that ED use may offer an early alert of a pregnant person’s higher future risk of severe adverse maternal and perinatal outcomes.

## Teaser

This cohort study examines the association between prepregnancy emergency department use and adverse maternal and perinatal outcomes.

## Introduction

Within most industrialized nations, preconception and first trimester pregnancy care are typically provided by an individual’s family physician.^1^ Second and third trimester care are then either continued by a family physician or assumed by a midwife or obstetrician.^2^ In the absence of a primary care physician, or with the development of an unforeseen, new-onset, or worsening health condition, unplanned health care visits often default to the emergency department (ED).^3^

Individuals with a greater number of antecedent comorbidities tend to have more peripregnancy ED visits.^4^ Chronic conditions, such as obesity, diabetes, and hypertension, collectively affect 1 in 5 women of reproductive age, yet they often remain unrecognized or undertreated in the weeks preceding conception,^5,6^ a period that can directly inform the trajectory of the health of a pregnant person. US studies have also identified maternal and systemwide factors associated with ED use during pregnancy, including insufficient antenatal care, psychological comorbidity, low socioeconomic status, and substance use, with worse ensuing obstetrical outcomes.^7,8,9^ However, these US studies comprised either commercially insured or low-income patient populations, who may differ considerably from women who receive care within a universally insured health care system, like that in Canada. As another limitation, existing research did not explore prepregnancy ED use, which would, naturally, be exclusive of any condition or concern arising in an index pregnancy. Specifically, studying ED utilization in the 90 days before pregnancy may describe both the health state and health care access of an individual preceding the critical time period of embryonic and placental development. To our knowledge, existing research has also not explored early-pregnancy ED use and its relation to pregnancy outcomes. Finally, prior studies did not measure important maternal or perinatal outcomes that tend to arise after the second trimester, including severe maternal morbidity (SMM), maternal mortality, stillbirth, or severe neonatal morbidity (SNM).

The current study, undertaken within a Canadian health care system and where all residents receive universally insured care, examined the association between prepregnancy ED use and important adverse maternal and perinatal outcomes. These same outcomes were further assessed in relation to ED use within the first trimester of pregnancy.

## Methods

### Setting

This retrospective population-based cohort was completed within all of Ontario, Canada’s most populous province, with universal health care coverage and standardized collection of all outpatient, ED, and inpatient hospital care services. The use of data in this project was authorized under section 45 of Ontario’s Personal Health Information Protection Act, which does not require review by a Research Ethics Board or informed consent from participants. Reporting of the study findings was consistent with the Strengthening the Reporting of Observational Studies in Epidemiology (STROBE) checklist for observational cohort studies.

### Study Population

Included were all women aged 10 to 55 years with a hospital livebirth or stillbirth in Ontario between April 1, 2003, and January 31, 2020. Excluded were deliveries with an invalid Ontario Health Insurance Plan (OHIP) number, non-Ontario residents, multifetal births, deliveries before 20 weeks’ or after 42 weeks’ gestation, and liveborn infants discharged alive without a valid OHIP number (eFigure 1 in the Supplement).

### Data Sources

This study used validated health administrative databases for the entire province of Ontario and housed at ICES, including the Canadian Institute for Health Information Discharge Abstract Database (DAD), the Same Day Surgery Database and National Ambulatory Care Reporting System (NACRS) database, the OHIP claims database, and the ICES MOMBABY database, which links the DAD hospital admission records of delivering mothers and their newborns^10,11,12,13^ (eTable 1 in the Supplement). Residential income quintile and rural residence were based on Statistics Canada census data using the 6-digit maternal postal code.^14^ These data sets were linked using unique encoded identifiers and analyzed at ICES. Date of conception is estimated using the clinical gestational weeks at delivery on the hospital birth record, which is based on accurate pregnancy dating by first- or second-trimester ultrasound for more than 95% of births in Ontario.^15^

### Exposures and Outcomes

The main exposure was any prepregnancy ED encounter within 90 days preceding time zero, which was the estimated conception date of the index pregnancy minus 2 weeks. The reason for setting time zero to 0 weeks’ gestation (equivalent to the first day of an individual’s last menstrual period) was to account for any potential inaccuracy in pregnancy dating and to ensure that the individual was not yet pregnant. An ED is defined as a hospital facility that serves unscheduled patients whose conditions may require immediate care and must be staffed by a physician at all times. An ED visit is defined as an encounter in the ED between a patient seeking care and a physician or other health care professional (ie, physician assistant or nurse practitioner working under physician supervision). A secondary study exposure was any ED visit in the first trimester of pregnancy, between 0 to 12 completed weeks’ gestation, when ED use tends to peak.^4^

All ED visits were identified in the NACRS database, defined as an ED encounter between a patient seeking care and a physician or physician assistant or nurse practitioner working under a physician’s supervision (eTable 1 in the Supplement). ED encounters that resulted in an ED discharge or in a hospital admission were included.

The *primary maternal study outcome* was a composite of SMM arising from 20 weeks’ gestation up to 42 days’ post partum (eTable 1 in the Supplement). This SMM composite (like that used in the United States and by the World Health Organization) has been validated against maternal mortality and maternal hospital length of stay and comprises diagnoses and procedures reflective of 44 unique severe maternal conditions, such as sepsis, severe preeclampsia, and major postpartum hemorrhage.^16,17,18^ A secondary maternal outcome was SMM or maternal death from 20 weeks’ gestation up to 42 days’ post partum.

A secondary *perinatal outcome* was a composite of SNM among all liveborn infants, arising from birth up to 27 days thereafter (eTable 1 in the Supplement). SNM, derived in Australia,^19^ and validated elsewhere,^20^ comprises diagnoses and procedures reflective of a severely ill newborn, including sepsis, intraventricular hemorrhage, and respiratory distress syndrome. Other secondary perinatal outcomes included neonatal death from birth up to 27 days thereafter as well as a stillbirth occurring from 20 weeks’ gestation onward.

### Statistical Analysis

Baseline variables were contrasted using standardized differences, comparing women who did vs did not have an ED visit within 90 days before the index pregnancy. For a given variable, a standardized difference greater than 0.10 reflects an important difference between groups.^21^

For the main analyses, modified Poisson regression with a robust error variance^22^ was used to generate relative risks (RRs) and 95% CIs for the association between a participant having any vs no ED visit within 90 days before pregnancy and each respective study outcome. In the case of more than 1 pregnancy in the same participant during the study period, generalized estimating equations with an exchangeable correlation structure accounted for correlated errors. RRs were adjusted for maternal age, neighborhood income quintile, and neighborhood rurality. An additional analysis further added to the multivariable models the number of comorbidities at the start of the index pregnancy, expressed as the total number of Johns Hopkins ACG System Aggregated Diagnosis Groups (ADG; groups were ≤2, 3-4, 5-6, and 7-32). A priori, it was understood that the number of ADGs would likely be collinear with a participant’s likelihood of an ED visit before pregnancy and would markedly attenuate the observed RRs. Post hoc, we also stratified by the number of ADG groups (≤2, 3-4, 5-6, and 7-32) to assess whether the association between a prepregnancy ED visit and SMM or SNM was consistent across ADG groups.

Next, in a dose-response analysis, the risk of SMM and SNM were each evaluated in relation to the number of ED encounters in the 90-day prepregnancy period. The number of ED visits was categorized as 0 (reference group), 1, 2, or 3 or more visits, and otherwise modeled as in the main analyses.

In NACRS, each ED visit is assigned a “Main Problem” (ie, main diagnosis), which is grouped into one of the existing chapters of the *International Statistical Classification of Diseases and Related Health Problems, Tenth Revision, Canada* (*ICD-10-CA*).^23^ Accordingly, the RR for SMM was also assessed in relation to the Main Problem at a participant’s latest (ie, most recent) ED visit within the 90-day prepregnancy period, relative to those with no ED visit (reference group). These results were further stratified by gravidity (nulligravid vs gravid) and by parity (nulliparous vs parous) preceding the index pregnancy. The median (IQR) number of days between the last prepregnancy ED visit and time zero were also calculated for each.

Analyses were conducted using SAS statistical software version 9.4 for Unix (SAS Institute) and the Johns Hopkins ACG System Version 10. Statistical significance was set at *P* < .05, and all tests were 2-tailed. All cell sizes of 1 to 5 were suppressed to prevent patient re-identification. A sample size calculation was not conducted prior to commencing this study, as all available births in the study period were included.

## Results

There were 2 255 940 livebirths and stillbirths identified, of which 125 695 births (5.6%) were excluded, owing to multifetal delivery, invalid OHIP number, duplicate delivery, extreme maternal age, or non-Ontario residency (eFigure 1 in the Supplement). Of the 2 130 245 included, 2 119 335 (99.5%) were a livebirth and 10 910 (0.5%) a stillbirth (eFigure 1 in the Supplement). The mean (SD) age of the cohort was 29.6 (5.4) years, 212 478 (9.9%) resided in a rural area, and 498 219 (23%) had 3 or more ADGs.

Among all births, 218 011 (9.7%) had an ED visit within 90 days before pregnancy, occurring at a median (IQR) of 48 days (26-69 days) preceding time zero. In contrast to those who did not visit the ED before pregnancy, women who visited the ED were more likely to be younger, reside in a lower income or rural area, and to have a greater number of ADGs (Table 1). Antenatal care was provided by an obstetrician in 975 953 of 1 912 234 pregnancies (73.0%) with no prepregnancy ED visit and 109 621 of 218 011 (71.1%) with a prepregnancy ED visit and did not differ by exposure groups.

**Table 1.** Baseline Characteristics of Participants With and Without an ED Visit Within 90 Days Before Pregnancy, or From 0 to 12 Weeks’ Gestation

Maternal characteristic	ED visit within 90 d before pregnancy			ED visit from 0 to 12 weeks’ gestation		
	Participants, No. (%)		Standardized difference^a^	Participants, No. (%)		Standardized difference^a^
	No (n = 1 912 234)	Yes (n = 218 011)		No (n = 1 763 085)	Yes (n = 367 160)	
At the start of the index pregnancy						
Age, y						
Mean (SD)	29.8 (5.3)	27.6 (5.9)	0.38	29.9 (5.3)	28.0 (5.8)	0.34
10-19	66 492 (3.5)	21 080 (9.7)	0.25	58 414 (3.3)	29 158 (7.9)	0.20
20-24	246 118 (12.9)	47 540 (21.8)	0.23	217 833 (12.4)	75 825 (20.7)	0.21
25-29	578 957 (30.3)	65 195 (29.9)	0.01	530 701 (30.1)	113 451 (30.9)	0.02
30-34	664 601 (34.8)	56 191 (25.8)	0.20	621 570 (35.3)	99 222 (27.0)	0.18
35-39	301 876 (15.8)	23 982 (11.0)	0.14	284 071 (16.1)	41 787 (11.4)	0.14
40-44	51 599 (2.7)	3869 (1.8)	0.06	48 106 (2.7)	7362 (2.0)	0.05
45-55	2591 (0.1)	154 (0.1)	0.02	2390 (0.1)	355 (0.1)	0.01
Residential income quintile						
1, Lowest	426 319 (22.3)	59 459 (27.3)	0.12	384 976 (21.8)	100 802 (27.5)	0.13
2	385 720 (20.2)	46 155 (21.2)	0.02	353 833 (20.1)	78 042 (21.3)	0.03
3	390 310 (20.4)	42 481 (19.5)	0.02	360 581 (20.5)	72 210 (19.7)	0.02
4	390 747 (20.4)	39 454 (18.1)	0.06	363 895 (20.6)	66 306 (18.1)	0.07
5, Highest	312 170 (16.3)	29 269 (13.4)	0.08	293 405 (16.6)	48 034 (13.1)	0.10
Missing	6968 (0.4)	1193 (0.5)	0.03	6395 (0.4)	1766 (0.5)	0.02
Rural residence	173 933 (9.1)	38 545 (17.7)	0.25	158 756 (9.0)	53 722 (14.6)	0.17
Gravidity, median (IQR), No.	1.0 (0.0-2.0)	1.0 (0.0-2.0)	0.20	1.0 (0.0-2.0)	1.0 (0.0-2.0)	0.03
Parity, median (IQR), No.	1.0 (0.0-1.0)	1.0 (0.0-1.0)	0.01	1.0 (0.0-1.0)	1.0 (0.0-1.0)	0.05
ADGs <120 d before pregnancy, No.^b^						
0-2	1 573 553 (82.3)	58 473 (26.8)	1.34	1 391 024 (78.9)	241 002 (65.6)	0.30
3-4	271 982 (14.2)	89 192 (40.9)	0.63	279 603 (15.9)	81 571 (22.2)	0.16
5-6	56 417 (3.0)	48 863 (22.4)	0.61	73 956 (4.2)	31 324 (8.5)	0.18
7-32	10 282 (0.5)	21 483 (9.9)	0.43	18 502 (1.0)	13 263 (3.6)	0.17
During the index pregnancy						
Antenatal care professional^c^						
Midwife	134 545 (10.1)	14 649 (9.5)	0.01	127 191 (10.4)	22 003 (8.4)	0.05
Obstetrician	975 953 (73.0)	109 621 (71.1)	0.02	892 385 (72.6)	193 189 (73.7)	0.04
Family physician or nurse practitioner	149 138 (11.2)	21 653 (14.0)	0.08	138 233 (11.2)	32 558 (12.4)	0.04
Other	4395 (0.3)	587 (0.4)	0.01	4086 (0.3)	896 (0.3)	0.00
None	7435 (0.6)	895 (0.6)	0.00	7044 (0.6)	1286 (0.5)	0.01
Unknown	65 226 (4.9)	6858 (4.4)	0.01	59 932 (4.9)	12 152 (4.6)	0.00
At the index birth						
Livebirth	1 902 604 (99.5)	216 731 (99.4)	0.01	1 754 592 (99.5)	364 743 (99.3)	0.02
Stillbirth	9630 (0.5)	1280 (0.6)		8493 (0.5)	2417 (0.7)	
Gestational at birth, mean (SD), wk^d^	38.9 (1.8)	38.7 (2.0)	0.07	38.9 (1.8)	38.6 (2.1)	0.15
Preterm <37 weeks’ gestation	115 619 (6.1)	16 743 (7.7)	0.07	100 644 (5.7)	31 718 (8.7)	0.11

Abbreviations: ADG, Aggregated Diagnosis Group; ED, emergency department.

^a^ For a given variable, a standardized difference greater than 0.10 reflects an important difference between groups.

^b^ Using ADGs within the 120 days before the clinical start of the index pregnancy.

^c^ Restricted to 1 490 955 births in the Better Outcomes Registry & Network database from April 2006 to March 2018.

^d^ Restricted to 2 119 335 livebirths.

### Risk of Adverse Maternal Outcomes

Women who had a prepregnancy ED visit had a higher rate of SMM (22.3 per 1000) than those without an ED visit (16.5 per 1000), equivalent to an unadjusted RR of 1.34 (95% CI, 1.30-1.38), an adjusted RR (aRR) of 1.37 (95% CI, 1.33-1.42), and an adjusted risk difference of 6.0 (95% CI, 5.4-6.7) (Table 2). This risk was slightly more pronounced among parous than nonparous women (eTable 2 in the Supplement). The risk of SMM or death was also higher in prepregnancy ED users (Figure). Further adjusting for the number of ADGs prior to pregnancy attenuated the observed RR for SMM or death, which nevertheless remained significant (eFigure 2 in the Supplement). Rather than adjusting for the number of ADGs, and instead stratifying by ADG groups, the absolute risk of SMM rose with a higher number of ADGs, but the aRR remained similar to that in the main model (eTable 3 in the Supplement).

**Table 2.** Risk of SMM Arising From 20 Weeks’ Gestation to 42 Days’ Post Partum in Relation to a Pregnant Person Having an ED Visit Within 90 Days Preceding the Estimated Clinical Start of Pregnancy or From 0 to 12 Weeks’ Gestation

Timing of ED visit	Participant, No.	SMM events (rate per 1000)	Relative risk (95% CI)		Adjusted absolute risk difference (95% CI)^a^
			Unadjusted	Adjusted^a^	
**Within 90 d before the index pregnancy**					
No ED visit	1 912 234	31 559 (16.5)	1 [Reference]	1 [Reference]	0 [Reference]
ED visit	218 011	4853 (22.3)	1.34 (1.30-1.38)	1.37 (1.33-1.42)	6.0 (5.4-6.7)
**From 0 to 12 weeks’ gestation**					
No ED visit	1 763 085	27 874 (15.8)	1 [Reference]	1 [Reference]	0 [Reference]
ED visit	367 160	8538 (23.3)	1.46 (1.42-1.49)	1.50 (1.46-1.54)	7.5 (7.0-8.1)

Abbreviations: ED, emergency department, SMM, severe maternal mortality.

^a^ Adjusted for maternal age, neighborhood income quintile, and neighborhood rurality at the start of the index pregnancy.

**Figure.**
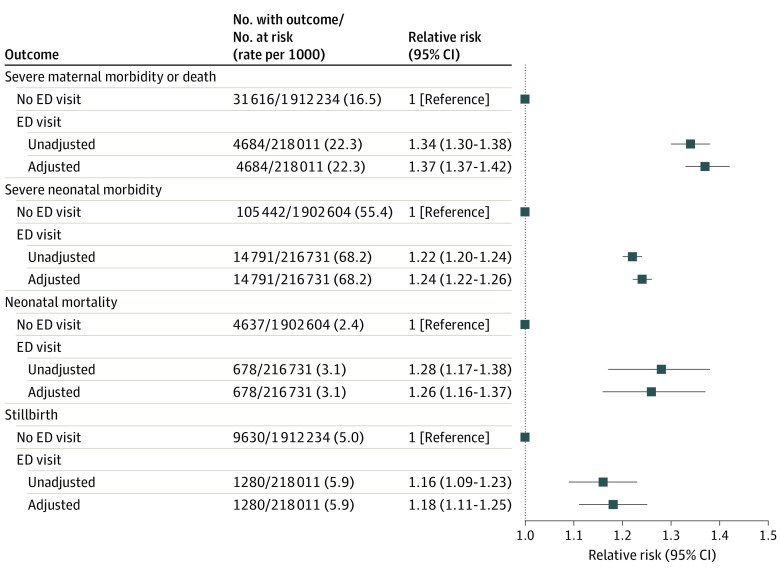
Risk of Severe Maternal Morbidity or Death, Severe Neonatal Morbidity, Neonatal Mortality, and Stillbirth This figure depicts the risk of the secondary study outcomes of severe maternal morbidity or death as well as severe neonatal morbidity, neonatal mortality, and stillbirth, each in relation to a woman having an emergency department (ED) visit within 90 days preceding the estimated clinical start of pregnancy. Shown are unadjusted relative risks, and relative risks adjusted for maternal age, neighborhood income quintile, and rurality. Whiskers denote 95% CIs.

A dose-response association was seen between the number of pre-pregnancy ED visits and the risk of SMM (Table 3). Compared with a participant without a prepregnancy ED visit, those with 1 visit had an aRR of 1.29 (95% CI, 1.24-1.34); 2 visits, 1.51 (95% CI, 1.42-1.61); and 3 or more visits, 1.74 (95% CI, 1.61-1.90).

**Table 3.** Risk of SMM Arising From 20 Weeks’ Gestation to 42 Days’ Post Partum and SNM Arising Less Than 28 Days After a Livebirth in Relation to the Number of ED Visits Within 90 Days Preceding the Estimated Clinical Start of Pregnancy

ED visits, No.	Births, No.	Events (rate per 1000)	Relative risk (95% CI)	
			Unadjusted	Adjusted^a^
**Outcome of SMM**				
0	1 912 234	31 559 (16.5)	1 [Reference]	1 [Reference]
1	155 375	3253 (20.9)	1.26 (1.21-1.30)	1.29 (1.24-1.34)
2	42 653	1038 (24.3)	1.46 (1.37-1.55)	1.51 (1.42-1.61)
≥3	19 983	562 (28.1)	1.67 (1.54-1.82)	1.74 (1.61-1.90)
**Outcome of SNM**				
0	1 902 604	105 442 (55.4)	1 [Reference]	1 [Reference]
1	154 500	10 195 (66.0)	1.18 (1.16-1.21)	1.20 (1.17-1.22)
2	42 379	2976 (70.2)	1.26 (1.21-1.30)	1.28 (1.23-1.32)
≥3	19 852	1620 (81.6)	1.45 (1.38-1.52)	1.48 (1.41-1.55)

Abbreviations: ED, emergency department, SMM, severe maternal morbidity; SNM, severe neonatal morbidity.

^a^ Adjusted for maternal age, neighborhood income quintile and rurality at the start of the index pregnancy.

Each of the main diagnostic groups at the latest prepregnancy ED visit was associated with a higher risk of SMM, with the highest aRRs seen for diseases of the blood (aRR, 13.60; 95% CI, 10.48-17.64); endocrine, nutritional, and metabolic systems (aRR, 4.96; 95% CI, 3.72-6.62); and the circulatory system (aRR, 2.27; 95% CI, 1.68-3.07) (Table 4). Of note, 40 891 women (18.8%) had a pregnancy-related condition diagnosed at their latest ED visit, occurring at a median of 53 days (IQR, 32-70 days) before the index pregnancy. Approximately 70% of these pregnancy-related conditions were for an abortive outcome and 25% for other maternal disorders related to pregnancy (Table 4). These associations persisted but varied slightly among nulligravid and gravid women or nulliparous and parous women (eTable 4 in the Supplement).

**Table 4.** Risk of Severe Maternal Morbidity Arising From 20 Weeks’ Gestation to 42 Days’ Post Partum in Relation to the Main Discharge Diagnosis Group at the Most Recent ED Visit Within the 90-Day Period Preceding the Estimated Clinical Start of Pregnancy

Main discharge diagnosis group (*ICD-10-CA grouping*)	Time before pregnancy, median (IQR), d	Participants affected, No.	Relative risk (95% CI)	
			Unadjusted	Adjusted^a^
No ED visit within 90 d before pregnancy	NA	1 912 234	1 [Reference]	1 [Reference]
Diseases of blood and blood-forming organs (D50-D89)	39 (20-61)	239	13.69 (10.61-17.67)	13.60 (10.48-17.64)
Endocrine, nutritional, and metabolic diseases (E00-E90)	40 (19-64)	558	4.86 (3.65-6.47)	4.96 (3.72-6.62)
Diseases of circulatory system (I00-I99)	45 (22-67)	1064	2.32 (1.72-3.14)	2.27 (1.68-3.07)
Neoplasms (C00-D48)	45 (20-70)	257	1.61 (0.77-3.40)	1.55 (0.73-3.27)
Mental, behavioral, and neurodevelopmental disorders (F00-F99)	40 (19-63)	5917	1.59 (1.36-1.86)	1.71 (1.47-2.00)
Diseases of skin and subcutaneous tissue (L00-L99)	41 (19-65)	4869	1.46 (1.22-1.75)	1.49 (1.25-1.78)
Diseases of nervous system (G00-G99)	41 (20-64)	3309	1.45 (1.16-1.80)	1.47 (1.19-1.83)
Symptoms, signs, abnormal clinical and laboratory findings, NEC (R00-R99)	41 (19-64)	38 518	1.44 (1.35-1.54)	1.51 (1.41-1.61)
Infections and parasitic diseases (A00-B99)	41 (20-64)	6596	1.35 (1.15-1.59)	1.41 (1.20-1.66)
Diseases of genitourinary system (N00-N99)	40 (19-64)	23 833	1.34 (1.23-1.46)	1.41 (1.29-1.53)
Diseases of musculoskeletal system (M00-M99)	41 (20-65)	9398	1.34 (1.17-1.53)	1.35 (1.18-1.54)
Diseases of respiratory system (J00-J99)	41 (20-65)	19 171	1.33 (1.21-1.46)	1.36 (1.23-1.49)
Injury, poisonings, and consequences of external causes (S00-T98)	42 (21-66)	33 609	1.32 (1.23-1.42)	1.38 (1.28-1.48)
Diseases of digestive system (K00-K93)	41 (20-64)	10 313	1.29 (1.13-1.47)	1.33 (1.17-1.52)
Pregnancy (O00-O99)^b^	53 (32-70)	40 891	1.11 (1.03-1.19)	1.11 (1.03-1.19)
Other or unknown	42 (21-66)	19 469	1.20 (1.08-1.32)	1.22 (1.10-1.35)

Abbreviation: ED, emergency department; *ICD-10-CA*, *International Classification of Diseases, 10th Revision, Canada*; NA, not applicable.

^a^ Adjusted for maternal age, neighborhood income quintile, and rurality at the start of the index pregnancy.

^b^ Among 40 891 ED visits (19%) in the 90 days before pregnancy that had a pregnancy-related *ICD-10-CA* code at the most recent ED visit, the majority of diagnoses (70%) were for an abortive outcome (*ICD-10-CA* code O00-O08) and 25% for other maternal disorders predominantly related to pregnancy (*ICD-10-CA* code O20-O29).

A total of 367 160 women had had an ED visit in the first trimester of pregnancy. This was also associated with a higher risk of SMM, compared with those with no ED encounter during that time (aRR, 1.50; 95% CI, 1.46-1.54) (Table 2).

There were 68 maternal deaths in the entire cohort (3.2 per 100 000 births). Of these, 11 (16.2%) had a prepregnancy ED visit.

### Risk of Adverse Perinatal Outcomes

The risk of SNM was higher among liveborn infants whose mother used the ED within 90 days before pregnancy (68.2 per 1000) than whose mother did not have an ED encounter (55.4 per 1000), for an aRR of 1.24 (95% CI, 1.22-1.26) (Figure). The same results were observed with respect to neonatal death (aRR, 1.26; 95% CI, 1.16-1.37) and stillbirth (aRR, 1.18; 95% CI, 1.11-1.25). Further adjusting for prepregnancy ADGs reduced the risk of each perinatal outcome, yet only neonatal death was no longer significant (eFigure 2 in the Supplement). Stratifying by ADG groups generated higher rates of SNM as the number of ADGs increased, with similar aRRs across strata (eTable 3 in the Supplement). A dose-response association was also seen between the number of prepregnancy ED visits and the risk of SNM (Table 3).

## Discussion

### Main Findings

Approximately 1 in 10 individuals who gave birth in Ontario had an ED visit within 90 days preceding the start of that index pregnancy, while 1 in 6 had an ED visit in the first trimester. Such ED use was associated with a higher risk for severe adverse maternal and perinatal outcomes arising later in pregnancy, especially as the number of ED visits increased.

### Other Studies

The current findings are consistent with prior research describing a positive association between the presence of maternal comorbidities and other socioeconomic factors and ED utilization.^3,4,8,24,25^ An ED visit during pregnancy is more likely among women younger than 25 years, nulliparous women, those residing in a low income or rural area, nonimmigrant women, and those with a greater number of ADGs.^4^ Rates of SMM in women with and without prepregnancy ED visits differed slightly in this Ontario population cohort than in some US studies,^26^ partly due to differences in the definition of SMM. For example, some US studies do not include major transfusion within the SMM composite and only evaluate SMM at the time of delivery, as opposed to up to 42 days thereafter.

In this study, nearly 19% of prepregnancy ED visits were for a pregnancy-related condition, presumably related to a prior pregnancy (Table 4). For example, the latest prepregnancy ED visit occurred at a median of 53 days before time zero, and approximately 70% of these visits were for some type of abortive condition. The observation that these women too had a higher risk of SMM underscores prior evidence that a short interpregnancy time interval is associated with adverse maternal and perinatal outcomes in some,^27,28^ but not all,^29^ studies. Moreover, women with recurrent miscarriage and infertility problems—often associated with polycystic ovarian syndrome and endometriosis—have a higher prevalence of chronic conditions, such as obesity, diabetes, and hypertension.^30^ Although less prevalent, prepregnancy ED visits for a hematological, endocrine, or circulatory condition was associated with the highest RR of SMM (Table 4). Prepregnancy anemia,^31^ diabetes,^32^ and chronic hypertension^33^ have congruently been shown to be associated with SMM, stillbirth, and neonatal mortality in large cohort studies. ED visits for mental health diagnoses were prevalent and, similar to other studies,^34,35^ were associated with a higher risk of serious adverse maternal and perinatal outcomes.

The current study introduces novel information about prepregnancy ED use and adverse perinatal outcomes. With ascertainment of the study exposure prior to the onset of pregnancy, the possibility of reverse causation was very low. In a retrospective cohort study of 107 207 Medicaid recipients in North Carolina who had a livebirth, 58% sought ED care 1 or more times during pregnancy, which was associated with a higher rate of preterm birth.^24^ A retrospective cohort study from Utah found that 3.9% of women experienced an injury-related ED visit during pregnancy, associated with an odds ratio of 1.23 (95% CI, 1.12-1.34) for preterm birth.^25^ In the current study, prepregnancy ED use showed a higher risk of SNM among both nulliparous and parous mothers, and we observed a dose-response association.

### Relevance to Clinical Practice and Policy

ED use in pregnancy is related to insufficient antenatal care, social instability, and higher existing comborbidities.^4,8,24^ Yet, even after adjusting for maternal age, neighborhood income and rurality, prepregnancy and first-trimester ED use remained associated with SMM and SNM. This is underscored by the fact that, across all diagnostic categories, ED utilization was associated with worse outcomes for mother and fetus, and especially, with a higher number of antecedent ED visits. Post hoc, higher rates of SMM and SNM were seen as the number of ADG-denoted comorbidities before pregnancy increased, even though the aRRs were similar (eTable 3 in the Supplement). Certainly, one need not posit that ED visits cause SMM or SNM; rather, ED utilization may be a red flag for insufficient care and/or suboptimally controlled disease. Further study is needed to determine whether any type of ED visit or ED visits for specific conditions are associated with adverse maternal and perinatal outcomes. Further comparison of ED encounters leading to hospital admission with those without admission might better reflect ED severity and the subsequent risk of SMM or SNM.

Efforts to improve preconception care should elaborate why so many women of reproductive age access the ED and what their needs are. Among women with chronic noncommunicable conditions, existing studies suggest a proactive approach of routinely asking those women about their pregnancy intentions and providing both advice on how to avoid unplanned pregnancy and specific recommendations about how to optimize their health once they wish to conceive. These women also want advice from peers with a similar health condition, including through online and social media.^36^ The latter is underscored by evidence from cluster randomized clinical trials completed in low- and middle-income countries, showing a 16% relative risk reduction (95% CI, 6%-25%) in perinatal mortality from group-based interventions that link prepregnancy with pregnancy care.^37^

Initiatives are also needed that enable an individual of reproductive age or who has a potentially viable pregnancy to receive timely care within a primary care and/or specialty clinic, rather than the ED.^38^ Even so, as preconception care applies not only to those planning a pregnancy, but also those who could become pregnant, a prepregnancy ED visit may be an opportunity for positive intervention. One example is oral iron supplementation among those with anemia.^39^ For those with prepregnancy diabetes, a preconception care bundle is associated with improved maternal and perinatal outcomes.^32,40^ And in those with chronic hypertension in pregnancy, blood pressure control (<140 mm Hg systolic blood pressure and <90 mm Hg diastolic blood pressure) is associated with fewer serious maternal complications.^41^

### Limitations

This study has limitations. It only included pregnancies ending in hospital livebirth or stillbirth, so the association between ED use and miscarriage or induced abortion was not assessed. ED use in pregnancy and the postpartum period has previously been observed to occur among 36% of livebirths, 47% of stillbirths, 74% of miscarriages, and 85% of threatened abortions.^4^

Details were lacking about the severity of participants’ conditions at their ED presentation or the pattern of care they received within the ED or thereafter. Whether a pregnancy was planned was not known, and deliveries occurring outside of a hospital setting were not captured, such as midwifery homebirths, which comprise fewer than 1% of livebirths in Ontario.^16^ We did not have information about race or ethnicity, which were not controlled for, and which have been shown to influence pregnancy-related ED utilization.^7^ Prior US studies correlated low socioeconomic status and substance use with worse pregnancy outcomes,^7,8,9^ but we could not account for smoking or other substance use.^42^ While the current study preceded the SARS-CoV-2 pandemic, fluctuations in ED use that may have been seen during the pandemic appear to be returning to prepandemic levels.^43,44^

By setting time zero at 2 weeks before conception, we ensured that each prepregnancy ED encounter was exclusive of the index pregnancy. As the latest ED visit occurred 48 days (IQR, 26-69 days) before the start of the pregnancy, this was likely true. Pregnancy dating in Ontario is likely to be accurate for most women who give birth.^15^ The fact that this study included all women in Ontario afforded care under a single-payer health care system likely reduced participant selection bias and information bias. Despite adjusting for certain maternal factors, and further adjusting for antecedent comorbidities, the potential remains for residual confounding between prepregnancy ED use and adverse maternal and perinatal outcomes, as discussed previously in the Discussion section.

## Conclusions

In this study, ED utilization was common both in the prepregnancy period and in early pregnancy. ED use may not only reflect an individual’s access to prepregnancy care but may offer a pragmatic early alert about an individual’s higher risk of SMM and SNM. Future studies could assess whether ED use is a useful trigger for health system interventions designed to decrease adverse pregnancy outcomes.

## Notes

Supplement. **eTable 1. **Variables Used to Define Cohort Entry and Exclusion Criteria, Outcomes, and Adjustment Variables **eTable 2. **Risk of Severe Maternal Morbidity From 20 Weeks’ Gestation to 42 Days Post Partum and Severe Neonatal Morbidity Arising Less Than 28 Days After a Livebirth in Relation to a Woman Having an Emergency Department (ED) Visit Within 90 Days Preceding the Estimated Clinical Start of Pregnancy, Further Stratified by Parity **eTable 3. **Post Hoc Analysis of the Risk of Severe Maternal Morbidity From 20 Weeks’ Gestation to 42 Days’ Post Partum and Severe Neonatal Morbidity Arising Less Than 28 Days After a Livebirth in Relation to a Woman Having an Emergency Department (ED) Visit Within 90 Days Preceding the Estimated Clinical Start of Pregnancy, Stratified by the Total Number of Aggregated Diagnosis Groups (ADGs) Within 120 Days Before the Estimated Clinical Start of Pregnancy **eTable 4. **Risk of Severe Maternal Morbidity Arising From 20 Weeks’ Gestation to 42 Days’ Post Partum in Relation to the Main Discharge Diagnosis Group at the Most Recent Emergency Department (ED) Visit Preceding the Estimated Clinical Start of Pregnancy **eFigure 1. **Flow Diagram of Cohort Creation **eFigure 2. **Risk of the Secondary Study Outcomes of Severe Maternal Morbidity or Death, Severe Neonatal Morbidity, Neonatal Mortality, and Stillbirth in Relation to A Woman Having an Emergency Department (ED) Visit Within 90 Days Preceding the Estimated Clinical Start of Pregnancy
