# Supplementary material for: Prepregnancy Emergency Department Use and Risks of Severe Maternal and Neonatal Morbidity in Canada
Source: JAMA Netw Open. 2022 Sep 2;5(9):e2229532. doi: 10.1001/jamanetworkopen.2022.29532 (PMC9440393; doi:10.1001/jamanetworkopen.2022.29532)
Supplement: Supplement. — eTable 1. Variables Used to Define Cohort Entry and Exclusion Criteria, Outcomes, and Adjustment Variables eTable 2. Risk of Severe Maternal Morbidity From 20 Weeks’ Gestation to 42 Days Post Partum and Severe Neonatal Morbidity Arising Less Than 28 Days After a Livebirth in Relation to a Woman Having an Emergency Department (ED) Visit Within 90 Days Preceding the Estimated Clinical Start of Pregnancy, Further Stratified by Parity eTable 3. Post Hoc Analysis of the Risk of Severe Maternal Morbidity From 20 Weeks’ Gestation to 42 Days’ Post Partum and Severe Neonatal Morbidity Arising Less Than 28 Days After a Livebirth in Relation to a Woman Having an Emergency Department (ED) Visit Within 90 Days Preceding the Estimated Clinical Start of Pregnancy, Stratified by the Total Number of Aggregated Diagnosis Groups (ADGs) Within 120 Days Before the Estimated Clinical Start of Pregnancy eTable 4. Risk of Severe Maternal Morbidity Arising From 20 Weeks’ Gestation to 42 Days’ Post Partum in Relation to the Main Discharge Diagnosis Group at the Most Recent Emergency Department (ED) Visit Preceding the Estimated Clinical Start of Pregnancy eFigure 1. Flow Diagram of Cohort Creation eFigure 2. Risk of the Secondary Study Outcomes of Severe Maternal Morbidity or Death, Severe Neonatal Morbidity, Neonatal Mortality, and Stillbirth in Relation to A Woman Having an Emergency Department (ED) Visit Within 90 Days Preceding the Estimated Clinical Start of Pregnancy [file jamanetwopen-e2229532-s001.pdf]

Supplemental Online Content

Varner CE, Park AL, Ray JG. Prepregnancy emergency department use and risks of severe maternal and neonatal morbidity in Canada. *JAMA Netw Open.* 2022;5(9):e2229532. doi:10.1001/jamanetworkopen.2022.29532

eTable 1. Variables Used to Define Cohort Entry and Exclusion Criteria, Outcomes, and Adjustment Variables

eTable 2. Risk of Severe Maternal Morbidity From 20 Weeks' Gestation to 42 Days Post Partum and Severe Neonatal Morbidity Arising Less Than 28 Days After a Livebirth in Relation to a Woman Having an Emergency Department (ED) Visit Within 90 Days Preceding the Estimated Clinical Start of Pregnancy, Further Stratified by Parity

eTable 3. Post Hoc Analysis of the Risk of Severe Maternal Morbidity From 20 Weeks' Gestation to 42 Days' Post Partum and Severe Neonatal Morbidity Arising Less Than 28 Days After a Livebirth in Relation to a Woman Having an Emergency Department (ED) Visit Within 90 Days Preceding the Estimated Clinical Start of Pregnancy, Stratified by the Total Number of Aggregated Diagnosis Groups (ADGs) Within 120 Days Before the Estimated Clinical Start of Pregnancy

eTable 4. Risk of Severe Maternal Morbidity Arising From 20 Weeks' Gestation to 42 Days' Post Partum in Relation to the Main Discharge Diagnosis Group at the Most Recent Emergency Department (ED) Visit Preceding the Estimated Clinical Start of Pregnancy

eFigure 1. Flow Diagram of Cohort Creation

eFigure 2. Risk of the Secondary Study Outcomes of Severe Maternal Morbidity or Death, Severe Neonatal Morbidity, Neonatal Mortality, and Stillbirth in Relation to A Woman Having an Emergency Department (ED) Visit Within 90 Days Preceding the Estimated Clinical Start of Pregnancy

This supplemental material has been provided by the authors to give readers additional information about their work.

© 2022 Varner CE et al. *JAMA Network Open.*

eTable 1. Variables Used to Define Cohort Entry and Exclusion Criteria, Outcomes, and Adjustment Variables				
**Assessment**	**Timing**	**Disease, procedure or measure**	**ICD-10-CA diagnostic codes and CCI procedure codes [bolded], in DAD, SDS and NACRS**	**ICD-9 diagnostic codes in OHIP {or other sources}**
Cohort entry criteria	At the index delivery date (where the estimated clinical start of pregnancy [i.e. 0 weeks' gestation] is April 1, 2003 to January 31, 2020)	All obstetrically delivered mothers and their linked newborns (livebirths and stillbirths) in the province of Ontario	Main patient service code indicating "obstetrical delivery" (the MOMBABY dataset includes linked DAD inpatient admission records of delivering mothers and their newborns; https://datadictionary.ices.on.ca/Applications/DataDictionary/Library.aspx?Library=MOMBABY)	--
Exclusion criteria	From 0 weeks' gestation up to and including 42 days' postpartum	Woman had an invalid healthcare number or hospital number	Invalid healthcare number on the DAD delivery record, or invalid maternal-newborn linkage in MOMBABY	Sex not 'Female', birth date missing, or unregistered healthcare number {RPDB}
	Same as above	Woman was a non-Ontario resident at any time during the perinatal period	--	Postal code {RPDB}
	Same as above	Woman was not OHIP eligible during the entire perinatal period	--	Eligibility start and end dates {RPDB}
	Estimated clinical start of pregnancy	Woman's recorded death date preceded pregnancy	--	Death date {RPDB}
	Same as above	Woman's age was missing or < 10 or > 55 years	--	Age {RPDB}

© 2022 Varner CE et al. *JAMA Network Open.*

	At the index birth	Length of gestation missing, < 20 weeks or > 42 weeks	Clinical gestation weeks at delivery (from the DAD newborn record, if present, otherwise from the DAD delivery record)	--
	Same as above	Multifetal delivery	Z37.2-Z37.7, Z37.90, O30, O31 (DAD delivery record) or Z38.3-Z38.6, Q89.4 (DAD newborn record)	--
	Same as above	Liveborn infant with unknown birthweight	Newborn weight (DAD newborn record)	--
	Same as above	Liveborn infant discharged alive and had an invalid healthcare number	Discharged alive and invalid healthcare number (DAD newborn record)	Sex missing, birth date missing, or unregistered healthcare number {RPDB}
Main Exposure	Within 90 days before the estimated clinical start of pregnancy	ED visit during the pre-pregnancy period	Any ICD-10-CA code in NACRS	--
Secondary Exposure	From the estimated clinical start of pregnancy up to 12 weeks' gestation	Main diagnostic code for the ED visit	Any ICD-10-CA code in NACRS	--
Main outcomes	Arising from 20 weeks' gestation up to 42 days after the index delivery	Severe Maternal Morbidity (SMM)	<u>Severe preeclampsia and hemolysis, elevated liver enzymes and low platelets (HELLP) syndrome</u>: O14.1, O14.2<br><br><u>Eclampsia</u>: O15<br><br><u>Cerebral venous thrombosis in pregnancy, or the puerperium</u>: O22.5, O87.3<br><br><u>Acute fatty liver with red blood cell (RBC) transfusion or plasma transfusion</u>: O26.6 + (CIHI-DAD RBC transfusion indicator=1 or CIHI-DAD Plasma transfusion indicator=1)<br><br><u>Pulmonary, cardiac, and CNS complications of anaesthesia during pregnancy, the puerperium, or labour and delivery</u>: O29.0, O29.1, O29.2, O89.0, O89.1, O89.2, O74.0, O74.1, O74.2, O74.3<br><br><u>Placenta previa with hemorrhage with RBC transfusion</u>: O44.1 + CIHI-DAD RBC transfusion indicator=1	--

© 2022 Varner CE et al. *JAMA Network Open.*

			Placental abruption with coagulation defect: O45.0<br><br>Antepartum hemorrhage with coagulation defect: O46.0<br><br>Intrapartum hemorrhage with coagulation defect: O67.0<br><br>Intrapartum hemorrhage with RBC transfusion: O67 + CIHI-DAD RBC transfusion indicator=1<br><br>Rupture of the uterus with RBC transfusion, procedures to the uterus or hysterectomy: O71.0 or O71.1 + any of the following:<br>• CIHI-DAD RBC transfusion indicator=1, or<br>• (**1.RM.13, 1.KT.51, 5.PC.91.LA or 5.PC.91.HV**) + CIHI-DAD RBC transfusion indicator=1, or<br>• (**5.MD.60.RC, 5.MD.60.RD, 5.MD.60.KE, 5.MD.60.CB or 1.RM.89.LA**[c])**,** or<br>• **1.RM.87.LA-GX**<br><br>[c] NOTE: **1.RM.89.LA** is included only if codes **1.PL.74, 1.RS.74 or 1.RS.80** are NOT also present<br><br>Postpartum hemorrhage with RBC transfusion, procedures to the uterus or hysterectomy: O72 + any of the following:<br>• CIHI-DAD RBC transfusion indicator=1, or<br>• (**1.RM.13, 1.KT.51, 5.PC.91.LA or 5.PC.91.HV**) + CIHI-DAD RBC transfusion indicator=1, or<br>• (**5.MD.60.RC, 5.MD.60.RD, 5.MD.60.KE, 5.MD.60.CB or 1.RM.89.LA**[d]), or<br>• **1.RM.87.LA-GX**<br><br>[d] NOTE: **1.RM.89.LA** is included only if codes **1.PL.74, 1.RS.74 or 1.RS.80** are NOT also present<br><br>Cardiac conditions: O74.2, O89.1, O90.3, I21, I22, I42, I43, I46, I49.0, I50, J81, **1.HZ.09, 1.HZ.30**<br><br>Obstetric shock: O75.1, R57, T80.5, T88.6<br><br>Septicemia during labour: O75.3<br><br>Complications of obstetric surgery and procedures: O75.4	

© 2022 Varner CE et al. *JAMA Network Open.*

			Puerperal sepsis: O85<br><br>Obstetric embolism: O88<br><br>Acute renal failure: O90.4, N17, N19, N99.0<br><br>Disseminated intravascular coagulation: D65<br><br>Sickle cell anemia with crisis: D57.0<br><br>Acute psychosis: F53.1, F23<br><br>Status epilepticus: G41<br><br>Cerebral edema or coma: G93.6, R40.2<br><br>Cerebrovascular diseases: subarachnoid and intracranial hemorrhage, cerebral infarction, stroke: I60, I61, I62, I63, I64<br><br>Status asthmaticus: J45.01, J45.11, J45.81, J45.91<br><br>Adult respiratory distress syndrome: J80<br><br>Acute abdomen: K35, K37, K65, N73.3, N73.5<br><br>Hepatic failure: K71, K72<br><br>Assisted ventilation through endotracheal tube: **1.GZ.31.CA-ND**<br><br>Assisted ventilation through tracheostomy: **1.GZ.31.CR-ND**<br><br>Hysterectomy: **5.MD.60.RC, 5.MD.60.RD, 5.MD.60.KE, 5.MD.60.CB, 1.RM.89.LA** (exclude if **1.PL.74, 1.RS.74 or 1.RS.80** code also present) **or 1.RM.87.LA-GX**<br><br>Dialysis: **1.PZ.21**<br><br>Evacuation of incisional hematoma with RBC transfusion: **5.PC.73.JS** + CIHI-DAD RBC transfusion indicator=1<br><br>Repair of bladder, urethra, or intestine: **5.PC.80.JR, 1.NK.80, 1.NM.80**	

© 2022 Varner CE et al. *JAMA Network Open.*

			<u>Procedures to the uterus/pelvic vessels with RBC transfusion</u>: (**1.RM.13, 1.KT.51, 5.PC.91.LA or 5.PC.91.HV**) + CIHI-DAD RBC transfusion indicator=1<br><br><u>Surgical or manual correction of inverted uterus for vaginal births only</u>: **5.PC.91.HQ or 5.PC.91.HP**, restricted to vaginal births (i.e., absence of caesarean **5.MD.60**)<br><br><u>Reclosure of caesarean wound</u>: (**5.PC.80.JM or 5.PC.80.JH**) + CIHI-DAD RBC transfusion indicator=1<br><br><u>Curettage with RBC transfusion</u>: (**5.PC.91.GA, 5.PC.91.GC or 5.PC.91.GD**) + CIHI-DAD RBC transfusion indicator=1<br><br><u>Maternal ICU admission</u>: CIHI-DAD Special Care Unit 10, 20, 25, 30, 35, 40, 45, 60, 80	
	Same as above	All-cause maternal death	--	Death date {RPDB}
	Arising < 28 days after a livebirth	Severe neonatal morbidity (SNM), using the severe neonatal adverse outcomes indicator (NAOI)	**All indicators are identified from the DAD newborn record:**<br><br><u>Gestational age at birth < 32 weeks</u><br><br><u>Birthweight < 1500 grams</u><br><br><u>Respiratory distress syndrome</u>: P22.0<br><br><u>Seizures</u>: P90, R56<br><br><u>Intraventricular haemorrhage (grades 3 and 4)</u>: P52.2<br><br><u>Cerebral infarction</u>: I63<br><br><u>Periventricular leukomalacia</u>: P91.2<br><br><u>Birth Trauma (intracranial hemorrhage paralysis due to brachial plexus injury, skull or long bone fracture)</u>: P10.0-3, P13.0, P13.2-3, P14.0-1<br><br><u>Hypoxic ischemic encephalopathy</u>: P91.5, P91.8, P91.6<br><br><u>Necrotising enterocolitis</u>: P77	Hypothermia (therapeutic) induction G210

© 2022 Varner CE et al. *JAMA Network Open.*

			Sepsis/septicaemia (streptococcus staphylococcus, E.coli, unspecified gram negative): P36.0-8, B95.1, B96.2<br><br>Pneumonia: P23, J12-18<br><br>Other respiratory (primary atelectasis, respiratory failure): P28.0, P28.5<br><br>Chronic respiratory disease originating in the perinatal period: P27<br><br>Bacterial meningitis: G00-03, G05<br><br>Resuscitation: **1.GZ.30.CJ, 1.GZ.30.CJ-NB, 1.GZ.30.JH, 1.HZ.30.JN, 1.HZ.30.JY**<br><br>Ventilatory support (mechanical ventilation and/or CPAP): **1.GZ.31.CA-EP, 1.GZ.31.CA-ND, 1.GZ.31.CA-PK, 1.GZ.31.CB-ND, 1.GZ.31.CR-ND, 1.GZ.31.GP-ND, 1.GZ.31.JA-GX, 1.GZ.31.JA-MD, 1.GZ.31.JA-NC, 1.GZ.31.JA-PK**<br><br>Central venous or arterial catheter: **1.KV.53.HA-CH, 1.KV.53.HA-FT, 1.KV.53.LA-FT, 2.IM.28.GP, 2.LZ.28.GQ-PL, 2.LZ.28.GR-PL, 2.LZ.28.JA-PL, 1.KX.53.HA-CH, 1.KX.53.HA-FT, 1.KX.53.LA-FT, 2.LZ.28.GQ-PL, 2.LZ.28.GR-PL**<br><br>Pneumothorax requiring intercostal catheter: P25.1, or **1.GV.52.DA, 1.GV.52.DA-TS, 1.GV.52.HA, 1.GV.52.HA-HE, 1.GV.52.HA-TK, 1.GV.52.LA, 1.GV.52.LA-TS, 1.GV.52.LA-XX-E, 1.GV.54.JA-TS, 1.GV.55.JA-TS**<br><br>Any intravenous fluids: **1.LZ.35.CA-E6, 1.LZ.35.HA-C1, 1.LZ.35.HA-C5, 1.LZ.35.HA-C6, 1.LZ.35.HA-C7, 1.LZ.35.HA-E6, 1.LZ.35.HA-T7, 1.LZ.35.HA-T9, 1.LZ.35.HA-Z9, 1.LZ.35.HH-C1, 1.LZ.35.HH-C5, 1.LZ.35.HH-C6, 1.LZ.35.HH-C7, 1.LZ.35.HH-E0, 1.LZ.35.HH-E6, 1.LZ.35.HH-T7, 1.LZ.35.HH-T9, 1.LZ.35.HH-Z9, 1.LZ.35.HR-C5, 1.LZ.35.HR-C6, 1.LZ.35.HR-C7, 1.LZ.35.HR-T9, 1.LZ.35.HR-Z9**<br><br>Any body cavity surgical procedure: **1.AA.52, 1.AA.87, 1.AC.87, 1.AE.87, 1.AF.87, 1.AG.87, 1.AJ.87, 1.AK.87, 1.AN.52, 1.AN.59, 1.AN.87, 1.AP.59, 1.AP.72, 1.AP.87, 1.AW.59, 1.AW.72, 1.AW.87, 1.AX.87, 1.BA.72, 1.BA.80, 1.BA.87, 1.BB.72, 1.BB.80, 1.BB.87, 1.BD.72, 1.BD.80, 1.BD.87, 1.BF.80, 1.BG.72, 1.BG.80, 1.BG.87, 1.BK.59,**	

© 2022 Varner CE et al. *JAMA Network Open.*

			**1.BM.72, 1.BM.80, 1.BM.87, 1.BN.72, 1.BN.80, 1.BN.87, 1.BP.72, 1.BP.80, 1.BP.87, 1.BQ.72, 1.BQ.80, 1.BQ.87, 1.BS.72, 1.BS.80, 1.BS.87, 1.BT.72, 1.BT.80, 1.BT.87, 1.GA.87, 1.GA.89, 1.GB.87, 1.GB.89, 1.GD.89, 1.GE.80, 1.GE.87, 1.GE.89, 1.GE.91, 1.GH.84, 1.GJ.86, 1.GJ.87, 1.GK.87, 1.GK.89, 1.GM.80, 1.GM.86, 1.GM.87, 1.GN.92, 1.GR.87, 1.GR.89, 1.GR.91, 1.GT.78, 1.GT.87, 1.GT.89, 1.GT.91, 1.GV.87, 1.GV.89, 1.GW.87, 1.GX.80, 1.GX.86, 1.GX.87, 1.GY.70, 1.GY.72, 1.GY.86, 1.HJ.76, 1.HJ.82, 1.HN.87, 1.HP.76, 1.HP.78, 1.HP.80, 1.HP.82, 1.HP.83, 1.HP.87, 1.HR.80, 1.HR.84, 1.HR.87, 1.HS.80 (excl. 1.HS.80.G), 1.HS.90, 1.HT.80 (excl. 1.HT.80.G), 1.HT.89, 1.HT.90, 1.HU.80 (excl. 1.HU.80.G), 1.HU.90, 1.HV.80 (excl. 1.HV.80.G), 1.HV.90, 1.HW.78, 1.HW.79, 1.HX.80, 1.HX.87, 1.HX.80, 1.HZ.87, 1.IA.76, 1.IA.80, 1.IA.87, 1.IB.76, 1.IB.79, 1.IB.80, 1.IB.82, 1.IB.87, 1.IC.76, 1.IC.80, 1.IC.82, 1.IC.87, 1.ID.76, 1.ID.80, 1.ID.82, 1.ID.86, 1.ID.87, 1.IF.83, 1.IJ.76, 1.IJ.80, 1.IM.76, 1.IM.80, 1.IM.82, 1.IM.83, 1.IM.87, 1.IN.83, 1.IN.84, 1.IN.87, 1.JE.57 (excl. 1.JE.57.G), 1.JE.76, 1.JE.80, 1.JE.87, 1.JJ.76, 1.JJ.80, 1.JK.76, 1.JK.80, 1.JK.87, 1.JW.51 (excl. 1.JW.51.G), 1.JW.57, 1.JW.76, 1.LA.84, 1.LC.84, 1.LD.84, 1.NA.72, 1.NA.74, 1.NA.76, 1.NA.77, 1.NA.80, 1.NA.84, 1.NA.86, 1.NA.87, 1.NA.88, 1.NA.89, 1.NA.90, 1.NA.91, 1.NA.92, 1.NE.80, 1.NF.76, 1.NF.78, 1.NF.80, 1.NF.82, 1.NF.84, 1.NF.86, 1.NF.87 (excl. 1.NF.87.B), 1.NF.89, 1.NF.90, 1.NF.91, 1.NF.92, 1.NK.76, 1.NK.77, 1.NK.80, 1.NK.82, 1.NK.84, 1.NK.87 (excl. 1.NK.87.B), 1.NM.74, 1.NM.76, 1.NM.77, 1.NM.80, 1.NM.82, 1.NM.87 (excl. 1.NM.87.B), 1.NM.89, 1.NM.91, 1.NP.72, 1.NP.73, 1.NP.86, 1.NQ.74 (excl. 1.NQ.74.B), 1.NQ.80, 1.NQ.84, 1.NQ.86, 1.NQ.87 (excl. 1.NQ.87.B), 1.NQ.89, 1.NQ.90, 1.NT.80, 1.NT.84, 1.NT.86, 1.NT.87, 1.NV.89, 1.OA.87, 1.OB.87, 1.OB.89, 1.OD.76, 1.OD.89, 1.OE.76, 1.OE.80, 1.OE.89, 1.OJ.76 (excl. 1.OJ.76.B), 1.OJ.87, 1.OJ.89, 1.OK.87, 1.OK.89, 1.OK.91, 1.OT.72, 1.OT.87, 1.OT.91, 1.PB.87, 1.PB.89, 1.PC.80, 1.PC.87 (excl. 1.PC.87.D), 1.PC.89, 1.PC.91, 1.PE.57 (excl. 1.PE.57.BD), 1.PE.80 (excl. 1.PE.80.D), 1.PE.82, 1.PE.87 (excl. 1.PE.87.D), 1.PE.89 (excl. 1.PE.89.D), 1.PG.76, 1.PG.77, 1.PG.80 (excl. 1.PG.80.D), 1.PG.86, 1.PG.89, 1.PL.74 (excl. 1.PL.74.CD), 1.PL.80, 1.PM.79, 1.PM.86, 1.PM.87 (excl. 1.PM.87.B), 1.PM.89, 1.PM.90, 1.PM.91, 1.PM.92, 1.QE.53, 1.QE.80, 1.QE.82, 1.QE.84, 1.QE.87, 1.QE.89, 1.QG.89, 1.QM.74, 1.QM.80, 1.QM.87, 1.QM.89, 1.QM.91, 1.QN.82, 1.QT.87, 1.QT.91, 1.RB.74, 1.RB.80, 1.RB.83, 1.RB.87, 1.RB.89, 1.RD.89, 1.RF.51, 1.RF.72, 1.RF.74, 1.RF.80, 1.RF.87, 1.RF.89, 1.RM.87 (excl. 1.RM.87.B), 1.RM.89, 1.RM.91, 1.RN.87, 1.RN.89, 1.RS.74, 1.RS.80, 1.RS.86, 1.RS.87, 1.RS.89, 1.RW.87, 1.RW.88, 1.RW.91, 1.RW.92, 1.SA.74, 1.SA.75, 1.SA.80, 1.SA.89, 1.SC.74, 1.SC.75, 1.SC.80, 1.SC.87, 1.SC.89, 1.SE.53, 1.SE.89 (excl. 1.SE.89.D), 1.SF.80, 1.SF.87, 1.SF.89, 1.SG.80, 1.SG.87, 1.SH.87, 1.SM.74, 1.SM.80,**	

© 2022 Varner CE et al. *JAMA Network Open.*

			**1.SM.87, 1.SN.87, 1.SN.93, 1.SQ.53, 1.SQ.74, 1.SQ.80, 1.SQ.87, 1.SQ.91, 1.SQ.93, 1.SW.74, 1.SY.80, 1.SY.84, 1.SY.87, 1.SZ.87, 1.VA.53, 1.VA.74, 1.VA.75, 1.VA.80, 1.VA.87, 1.VA.93, 1.VC.74, 1.VC.80, 1.VC.87, 1.VC.91, 1.VC.93, 1.VE.80, 1.VG.53, 1.VG.55, 1.VG.72, 1.VG.73, 1.VG.74, 1.VG.75, 1.VG.80, 1.VG.87, 1.VG.93, 1.VK.80, 1.VK.87, 1.VK.89, 1.VL.80, 1.VL.87, 1.VM.80, 1.VM.87, 1.VN.80, 1.VN.87, 1.VP.74, 1.VP.80, 1.VP.87, 1.VP.89, 1.VQ.74, 1.VQ.79, 1.VQ.80, 1.VQ.82, 1.VQ.87, 1.VQ.91, 1.VQ.93, 1.VS.72, 1.VS.80, 1.VX.87**	
	Same as above	All-cause neonatal death	Discharge disposition = 'Died' or 'Died in facility' (DAD newborn record)	Death date {RPDB}
	At the index delivery	Stillbirth at ≥ 20 weeks' gestation	Z37.1, Z37.3, Z37.4, Z37.6, Z37.7, O36.4 (DAD delivery record)	--
Covariates	At the estimated clinical start of pregnancy	Woman's age	--	Age {RPDB}
	Same as above	Woman's area-level income quintile based on her 6-digit postal code	--	{Statistics Canada census}
	Same as above	Woman's rural residence based on her 6-digit postal code	--	{Statistics Canada census}
	At the index delivery	Woman's number of previous deliveries	Previous term deliveries + Previous pre-term deliveries (DAD delivery record)	--
	Same as above	Woman's number of previous pregnancies	Previous term deliveries + Previous pre-term deliveries + Previous spontaneous abortions + Previous therapeutic abortions (DAD delivery record)	--
	Within 120 days before the estimated clinical start of pregnancy	Total number of Aggregated Diagnosis Groups (ADGs; 0-2, 3-4, 5-6, 7-32)	ADGs were obtained from diagnosis codes in DAD, SDS and NACRS using The Johns Hopkins ACG® System software	--

© 2022 Varner CE et al. *JAMA Network Open.*

	During the index pregnancy[a]	Antenatal care provider	--	{BORN}

ADG Aggregated Diagnosis Group; CCI Canadian Classification of Interventions; CCP Canadian Classification of Diagnoses and Procedures; ICD-9 International Classification of Diseases, 9th Revision; ICD-10-CA International Classification of Diseases, 10th Revision, Canada; NACRS National Ambulatory Care Reporting System; OHIP Ontario Health Insurance Plan; RPDB Registered Persons Database; SDS Same Day Surgery Database

[a] Restricted to deliveries in the Better Outcomes Registry & Network database from April 2006 to March 2018.

© 2022 Varner CE et al. *JAMA Network Open.*

eTable 2. Risk of Severe Maternal Morbidity From 20 Weeks' Gestation to 42 Days Post Partum and Severe Neonatal Morbidity Arising Less Than 28 Days After a Livebirth in Relation to a Woman Having an Emergency Department (ED) Visit Within 90 Days Preceding the Estimated Clinical Start of Pregnancy, Further Stratified by Parity

				Relative risk (95% confidence interval)	
**Outcome**	**Parity**	**ED visit within 90 days before pregnancy**	**Number of outcome events (rate per 1000)**	**Unadjusted**	**Adjusted[a]**
**Severe maternal morbidity**	*Nulliparous*	No ED visit (N = 836,847)	16,553 (19.8)	1.00 (ref.)	1.00 (ref.)
		ED visit (N = 97,943)	2432 (24.8)	1.26 (1.20 to 1.31)	1.33 (1.27 to 1.38)
	Parous	No ED visit (N = 1,075,109)	15,003 (14.0)	1.00 (ref.)	1.00 (ref.)
		ED visit (N = 120,027)	2420 (20.2)	1.43 (1.37 to 1.50)	1.48 (1.42 to 1.55)
**Severe neonatal morbidity**	*Nulliparous*	No ED visit (N = 832,252)	55,613 (66.8)	1.00 (ref.)	1.00 (ref.)
		ED visit (N = 97,347)	7365 (75.7)	1.13 (1.11 to 1.16)	1.18 (1.15 to 1.21)
	Parous	No ED visit (N = 1,070,078)	49,802 (46.5)	1.00 (ref.)	1.00 (ref.)
		ED visit (N = 119,343)	7424 (62.2)	1.32 (1.29 to 1.35)	1.35 (1.32 to 1.38)

[a]Adjusted for maternal age, neighbourhood income quintile and rurality at the start of the index pregnancy.

© 2022 Varner CE et al. *JAMA Network Open.*

eTable 3. Post Hoc Analysis of the Risk of Severe Maternal Morbidity From 20 Weeks' Gestation to 42 Days' Post Partum and Severe Neonatal Morbidity Arising Less Than 28 Days After a Livebirth in Relation to a Woman Having an Emergency Department (ED) Visit Within 90 Days Preceding the Estimated Clinical Start of Pregnancy, Stratified by the Total Number of Aggregated Diagnosis Groups (ADGs) Within 120 Days Before the Estimated Clinical Start of Pregnancy

				Relative risk (95% confidence interval)	
**Outcome**	**Pre-pregnancy ADG group**	**ED visit within 90 days before pregnancy**	**Number of outcome events (rate per 1000)**	**Unadjusted**	**Adjusted[a]**
**Severe maternal morbidity**	*0-2*	No ED visit (N = 1,573,553)	24,950 (15.9)	1.00 (ref.)	1.00 (ref.)
		ED visit (N = 58,473)	1129 (19.3)	1.21 (1.14 to 1.29)	1.24 (1.16 to 1.31)
	3-4	No ED visit (N = 271,982)	5140 (18.9)	1.00 (ref.)	1.00 (ref.)
		ED visit (N = 89,192)	1826 (20.5)	1.08 (1.03 to 1.14)	1.13 (1.07 to 1.19)
	5-6	No ED visit (N = 56,417)	1202 (21.3)	1.00 (ref.)	1.00 (ref.)
		ED visit (N = 48,863)	1226 (25.1)	1.18 (1.09 to 1.27)	1.22 (1.12 to 1.32)
	7-32	No ED visit (N = 10,282)	267 (26.0)	1.00 (ref.)	1.00 (ref.)
		ED visit (N = 21,483)	672 (31.3)	1.20 (1.04 to 1.38)	1.25 (1.08 to 1.44)
**Severe neonatal morbidity**	*0-2*	No ED visit (N = 1,565,852)	84,504 (54.0)	1.00 (ref.)	1.00 (ref.)
		ED visit (N = 58,160)	3559 (61.2)	1.13 (1.09 to 1.17)	1.15 (1.11 to 1.19)
	3-4	No ED visit (N = 270,501)	16,449 (60.8)	1.00 (ref.)	1.00 (ref.)
		ED visit (N = 88,685)	5795 (65.3)	1.07 (1.04 to 1.10)	1.10 (1.06 to 1.13)
	5-6	No ED visit (N = 56,044)	3733 (66.6)	1.00 (ref.)	1.00 (ref.)
		ED visit (N = 48,585)	3571 (73.5)	1.10 (1.05 to 1.15)	1.13 (1.08 to 1.19)
	7-32	No ED visit (N = 10,207)	756 (74.1)	1.00 (ref.)	1.00 (ref.)
		ED visit (N = 21,301)	1866 (87.6)	1.18 (1.09 to 1.28)	1.19 (1.09 to 1.29)

[a]Adjusted for maternal age, neighbourhood income quintile and rurality at the start of the index pregnancy.

© 2022 Varner CE et al. *JAMA Network Open.*

eTable 4. Risk of Severe Maternal Morbidity Arising From 20 Weeks' Gestation to 42 Days' Post Partum in Relation to the Main Discharge Diagnosis Group at the Most Recent Emergency Department (ED) Visit Preceding the Estimated Clinical Start of Pregnancy

Findings are stratified by nulligravid and gravid women (upper), and by nulliparous and parous women (lower).

	Nulligravid women				Gravid women		
**Main discharge diagnosis group (ICD-10CA grouping)**	**Median (IQR) no. days pre-pregnancy**	**No. women affected**	**Adjusted RR (95% CI)[a]**		**Median (IQR) no. days pre-pregnancy**	**No. women affected**	**Adjusted RR (95% CI)[a]**
No ED visit within 90 days before pregnancy	Not applicable	641,278	1.00 (ref.)		Not applicable	1,270,682	1.00 (ref.)
Diseases the blood & blood-forming organs (D50-D89)	42 (21-61)	66	16.08 (10.79-23.97)		39 (19-61)	173	13.59 (9.99-18.49)
Endocrine, nutritional & metabolic diseases (E00-E90)	42 (18-69)	183	4.12 (2.48-6.83)		38 (20-64)	375	5.76 (4.11-8.07)
Diseases the circulatory system (I00-I99)	47 (23-69)	300	3.11 (1.99-4.88)		45 (21-66)	764	1.97 (1.32-2.95)
Diseases of nervous system (G00-G99)	42 (21-65)	1005	1.70 (1.21-2.39)		41 (20-64)	2303	1.41 (1.07-1.88)
Mental, behavioral & neurodevelopmental disorders (F00-F99)	41 (19-64)	2115	1.63 (1.26-2.10)		39 (19-62)	3798	1.86 (1.53-2.27)
Diseases of digestive system (K00-K93)	40 (20-64)	3069	1.42 (1.15-1.77)		41 (20-65)	7244	1.35 (1.15-1.60)
Symptoms, signs, abnormal clinical & laboratory findings, NEC (R00-R99)	41 (19-65)	12,693	1.40 (1.26-1.57)		41 (19-64)	25,817	1.63 (1.51-1.77)
Pregnancy (O00-O99)[b]	48 (29-68)	1756	1.30 (0.97-1.74)		53 (32-70)	39,129	1.24 (1.15-1.34)
Infections & parasitic diseases (A00-B99)	40 (19-64)	2200	1.36 (1.04-1.77)		41 (20-65)	4395	1.51 (1.24-1.84)
Diseases of genitourinary system (N00-N99)	39 (18-63)	7847	1.31 (1.14-1.52)		41 (19-65)	15,983	1.52 (1.37-1.70)
Diseases of respiratory system (J00-J99)	42 (20-65)	5940	1.29 (1.09-1.52)		40 (20-65)	13,225	1.47 (1.31-1.65)
Diseases of skin & subcutaneous tissue (L00-L99)	41 (19-65)	1659	1.26 (0.92-1.72)		41 (19-64)	3210	1.69 (1.36-2.11)
Injury, poisonings & consequences of external causes (S00-T98)	42 (21-66)	12,108	1.28 (1.14-1.44)		42 (20-65)	21,498	1.46 (1.33-1.60)
Other or unknown	40 (19-64)	5223	1.24 (1.03-1.48)		43 (22-66)	14,241	1.29 (1.14-1.46)
Neoplasms (C00-D48)	41 (22-70)	71	0.71 (0.10-5.03)		47 (20-70)	186	2.03 (0.92-4.51)

© 2022 Varner CE et al. *JAMA Network Open.*

Main discharge diagnosis group (ICD-10CA grouping)	Nulliparous women				Parous women		
	Median (IQR) no. days pre-pregnancy	No. women affected	Adjusted RR (95% CI)[a]		Median (IQR) no. days pre-pregnancy	No. women affected	Adjusted RR (95% CI)[a]
No ED visit within 90 days before pregnancy	Not applicable	836,847	1.00 (ref.)		Not applicable	1,075,109	1.00 (ref.)
Diseases of blood & blood-forming organs (D50-D89)	39 (21-59)	97	17.34 (12.78-23.53)		40 (19-63)	142	12.48 (8.64-18.04)
Endocrine, nutritional & metabolic diseases (E00-E90)	41 (18-67)	261	5.54 (3.87-7.94)		37 (20-61)	297	4.57 (2.93-7.11)
Diseases of circulatory system (I00-I99)	47 (22-69)	423	2.24 (1.44-3.49)		44 (21-65)	641	2.44 (1.62-3.67)
Diseases of nervous system (G00-G99)	41 (21-64)	1369	1.72 (1.29-2.30)		41 (20-64)	1939	1.32 (0.95-1.84)
Infections & parasitic diseases (A00-B99)	40 (19-64)	2922	1.50 (1.21-1.86)		41 (20-65)	3673	1.41 (1.11-1.78)
Symptoms, signs, abnormal clinical & laboratory findings, NEC (R00-R99)	41 (19-64)	17,788	1.46 (1.33-1.60)		41 (19-64)	20,722	1.63 (1.49-1.79)
Mental, behavioral & neurodevelopmental disorders (F00-F99)	40 (19-64)	3132	1.49 (1.20-1.86)		39 (19-63)	2781	2.08 (1.66-2.60)
Diseases of respiratory system (J00-J99)	41 (19-65)	7719	1.36 (1.18-1.56)		40 (20-65)	11,446	1.49 (1.31-1.69)
Diseases of digestive system (K00-K93)	40 (19-64)	4163	1.34 (1.10-1.62)		41 (20-65)	6150	1.44 (1.20-1.73)
Diseases of musculoskeletal system (M00-M99)	41 (21-65)	3983	1.28 (1.05-1.56)		41 (20-64)	5411	1.49 (1.24-1.79)
Diseases of skin & subcutaneous tissue (L00-L99)	41 (20-65)	2233	1.28 (0.98-1.67)		41 (19-64)	2636	1.80 (1.41-2.29)
Diseases of genitourinary system (N00-N99)	40 (19-63)	11,532	1.30 (1.15-1.46)		41 (19-65)	12,298	1.58 (1.40-1.79)
Other or unknown	42 (21-65)	8558	1.21 (1.05-1.39)		43 (21-66)	10,906	1.29 (1.12-1.49)
Injury, poisonings & consequences of external causes (S00-T98)	42 (21-66)	16,502	1.23 (1.11-1.36)		42 (20-65)	17,104	1.56 (1.41-1.74)
Pregnancy (O00-O99)[c]	53 (32-70)	17,152	1.13 (1.02-1.24)		53 (32-70)	23,733	1.12 (1.01-1.24)
Neoplasms (C00-D48)	46 (20-72)	109	0.89 (0.23-3.54)		44 (21-69)	148	2.26 (0.93-5.49)

[a] Adjusted for maternal age, neighbourhood income quintile and rurality at the start of the index pregnancy

[b] Among ED visits in the 90 days before pregnancy that had a pregnancy-related ICD-10-CA code at the latest ED visit, the majority of diagnoses (69% of nulligravid and 70% of gravid women) were for an abortive outcome (ICD-10-CA O00-O08), and other maternal disorders predominantly related to pregnancy (27% of nulligravid and 25% of gravid women) (ICD-10-CA O20-O29).

[c] Among ED visits in the 90 days before pregnancy that had a pregnancy-related ICD-10-CA code at the latest ED visit, the majority of diagnoses (72% of nulliparous and 69% of parous women) were for an abortive outcome (ICD-10-CA O00-O08), and 25% for other maternal disorders predominantly related to pregnancy (ICD-10-CA O20-O29).

ICD-10-CA International Classification of Diseases, 10th Revision, Canada; RR relative risk; CI confidence interval.

© 2022 Varner CE et al. *JAMA Network Open.*

eFigure 1. Flow Diagram of Cohort Creation

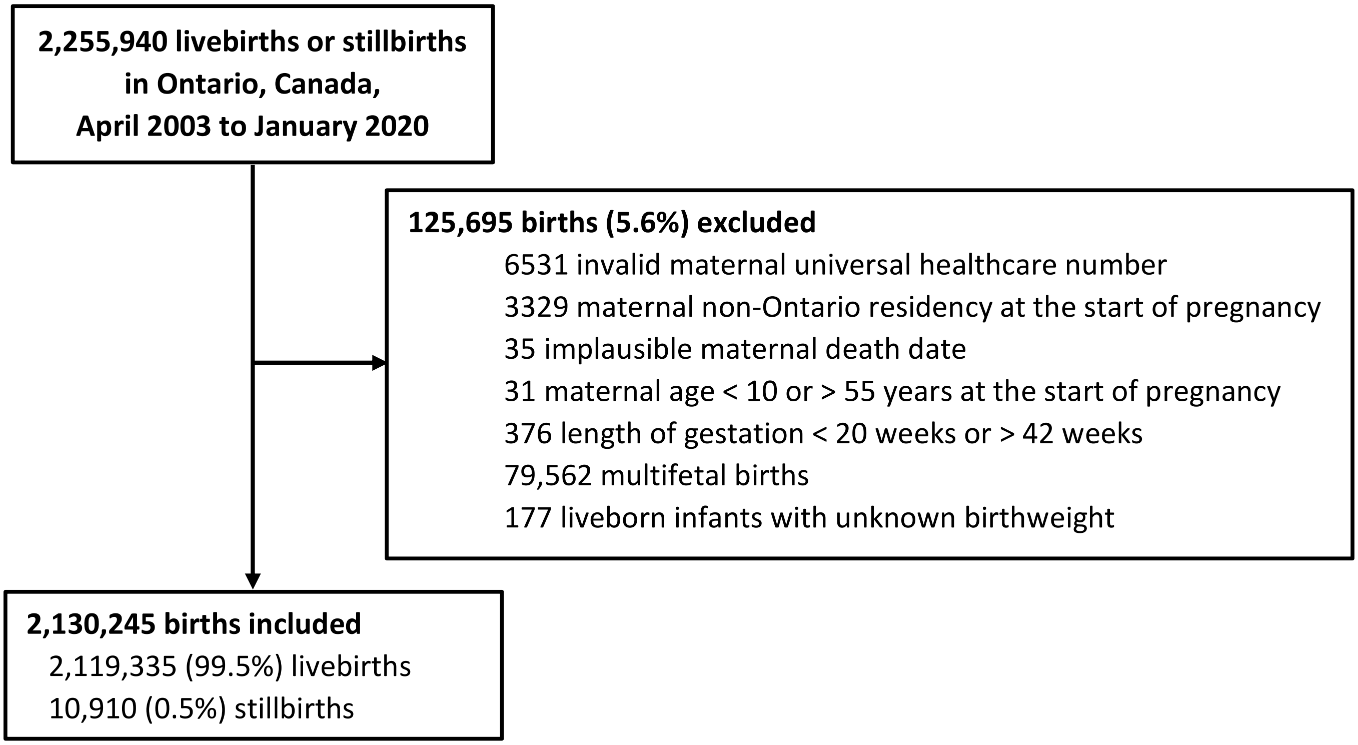

© 2022 Varner CE et al. *JAMA Network Open.*

eFigure 2. Risk of the Secondary Study Outcomes of Severe Maternal Morbidity or Death, Severe Neonatal Morbidity, Neonatal Mortality, and Stillbirth in Relation to A Woman Having an Emergency Department (ED) Visit Within 90 Days Preceding the Estimated Clinical Start of Pregnancy Shown are unadjusted relative risks (black squares), and relative risks adjusted for maternal age, neighbourhood income quintile, rurality, and total Aggregated Diagnosis Groups (red diamonds).

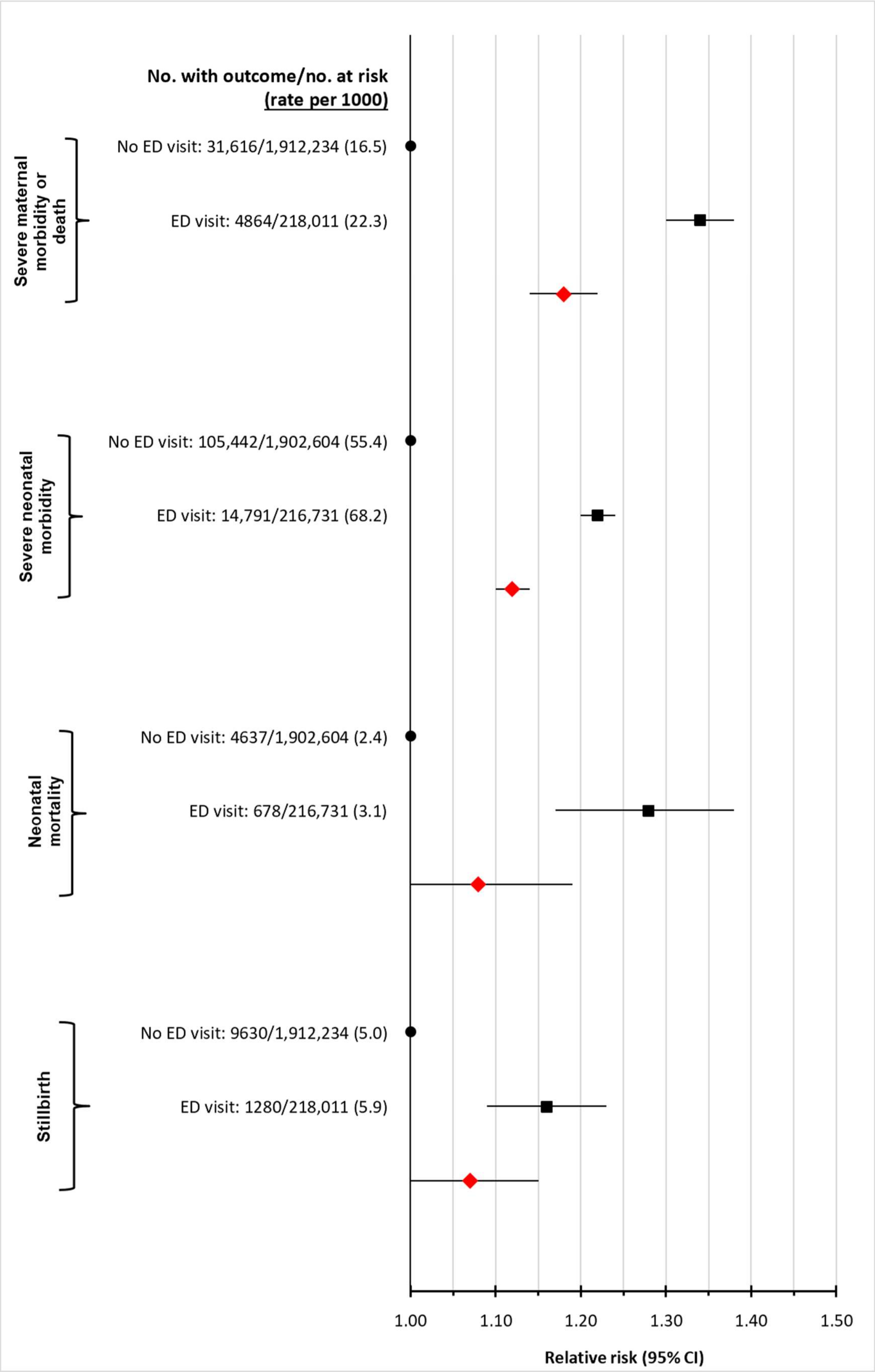

© 2022 Varner CE et al. *JAMA Network Open.*
